# Technique: A Word to Students

**Published:** 1898-04-30

**Authors:** 


### Technique: A Word to Students.

It may well be questioned whether surgeons spend as much time as they ought to do in studying the technique of their art. The painter is perhaps born with artistic instinct, but his power of expression by the brush is a matter of sheer practice. Who, again, who has lived next door to a violinist, a pianist, or even a singer, does not know what labour is expended by these artists in attaining perfection in apparently simple passages. Hours and hours of laborious practice do these people undergo before they attempt to perform in public, yet a surgeon who would be puzzled to mend his gloves, and would stand aghast at the thought of doing a little carpentering, enters with a light heart upon the work of operative surgery, involving the sawing of bones, the sewing of intestines, and the trephining of skulls.

Who ever sees a medical student spending hours practising the suturing of intestines? Yet, if he ever becomes a surgeon, how many lives may depend upon the nicety and accuracy with which it is done; and the same with every branch of the surgical technique. The success of certain surgeons is proverbial, or, as their colleagues have it, their luck is extraordinary. Luck, however, is a wide term, and many a surgeon is "lucky" because either from an inborn finger cleverness, or from careful and laborious practice, he gets through the technique of his work with professional exactitude; while many another is unlucky because he practises only on his patients and leaves on all his work the trail of the amateur. Nothing can be more striking than the differences which exist between different men in technical skill. Look at two men putting on plaster of Paris bandages. Look at the mess and the waste and the crackly product in the one case, and the hard, firm bandage, the effectiveness of each turn, and the cleanliness of the whole process, in the hands of the more skilful performer. Again, in abdominal operations, observe the apparent ease with which intestines come together in the hands of one surgeon and the lop-sidedness of the join in the hands of another. This needle work is by no means easy, and when we see the hours of practice which it takes to attain the art of hemming a pocket-handkerchief we have no right to believe that it will take less to learn the trick of putting in a Lembert so as not to use up half the gut in doing it, to do a "purse string" in such a manner that it shall pull together smoothly, or even to put in a continuous suture with just the right tension, so as, on the one hand, not to leave it gaping, and, on the other, not to pucker up the bowel into a half moon. We cannot too strongly insist upon the fact that these things do not come by instinct, and that only by patient practice can good technique be acquired. Suggestions have recently been made with the object of carrying out operative proceedings in a completely aseptic manner, which may indeed make it necessary for surgeons to train themselves still further in feats of manual skill. Whatever difficulty there may be in rendering the hands aseptic, there need be no difficulty at all in regard to metal instruments, and it is now suggested that if surgeons will operate by "the dry method," that is, without using any water or other lotion, and will keep their fingers from touching the wound, better results will be obtained even than are at present met with. Dr. Milton Foote, of New York, writing on this subject, says, "Most of the minor surgical operations, and many of the major ones, can be performed without direct contact between the fingers and the patient. After a little practice, ligatures and sutures may be tied with forceps or clamps, and when a man finishes an operation without a stain of blood on his fingers he is sure that he has not introduced germs from his hand into the wound. Quite recently I had the pleasure of assisting Dr. Walker in suturing a badly fractured patella. The joint had been widely opened, and contained clots of blood, though the skin was not torn. The blood clots were wiped away, the patella was sutured, and the incision was closed, neither of us having touched the wound or any part of any instrument or suture which came into contact with it."

Of course we know the old adage that "fingers were made before forks," and the implied suggestion that fingers are cleverer than all instrumental aids. Unfortunately, however, we cannot boil fingers; and, after all, to learn to use instruments is only a matter of time and trouble, as is the learning of technique in every art.

